# Fibre wall and lumen fractions drive wood density variation across 24 Australian angiosperms

**DOI:** 10.1093/aobpla/plt046

**Published:** 2013-10-10

**Authors:** Kasia Ziemińska, Don W. Butler, Sean M. Gleason, Ian J. Wright, Mark Westoby

**Affiliations:** 1 Department of Biological Sciences, Macquarie University, Sydney, NSW 2109, Australia; 2 Present address: Queensland Herbarium, Mt Coot-tha Road, Toowong, QLD 4066, Australia

**Keywords:** Ecological strategies, fibres, parenchyma, rays, tissue fraction/proportion/percentage/volume, vessels, wood anatomy.

## Abstract

Wood density is considered a key plant trait, affecting mechanical and physiological performance, yet its biological meaning is still rather unclear. Accordingly we investigated the anatomical underpinnings of wood density in trees and shrubs. We measured wood density and anatomical traits in distal stems 4–10 mm diameter under bark in 24 Australian species. Proportions of wood components that are functionally distinct were analysed, including fibre wall and lumen, vessel wall and lumen, and axial and ray parenchyma. Wood density was mainly driven by the density of wood outside vessel lumens (density_NV_) rather than by vessel lumen fraction. In turn, density_NV_ variation was chiefly affected by fibre wall and lumen fractions. Considerable anatomical variation was observed at a given density_NV_, especially among medium-density_NV_ species (0.60–0.85 g cm^−3^); this range of medium density_NV_ roughly translates to 0.50–0.75 g cm^−3^ of overall density. The anatomy of these species formed a continuum from low fibre lumen and medium parenchyma fractions to medium fibre lumen and low parenchyma fractions. Our data suggest that wood density is an emergent property influenced by a complex anatomy rather than an unambiguous functional trait, particularly in medium-density species. With much anatomical variation, they likely represent a wide range of ecological strategies.

## Abstract (precis)

Wood density is often considered to be a key plant functional trait. But it is not clear what actually wood density is? We rigorously quantified anatomical underpinnings of wood density variation. We found that density was mainly driven by properties of mechanical tissue such as fibre wall fraction and fibre lumen fraction. However, there was also a substantial anatomical variation independent of density. This variation suggests that different plant ecological strategies may be available to woods with the same density. Our results imply that density is a complex characteristic of wood rather than a straightforward indicator of plant ecological strategies.

## Introduction

Plants vary significantly in their ecological, physiological and mechanical properties or ‘traits’ both across climate and even within a site (Westoby *et al.* 2002; Wright *et al.* 2004; Chave *et al.* 2009). This indicates that there are multiple solutions to the problem of how to be a successful plant and maintain species continuity. Different solutions can be called plant ecological strategies (Westoby *et al.* 2002).

Wood density has been suggested as a key player in plant ecological strategies (Chave *et al.* 2009). Firstly, wood density has been linked with hydraulic strategies. Denser woods tend to operate at more negative water potentials (Ackerly 2004; Bucci *et al.* 2004; Santiago *et al.* 2004; Jacobsen *et al.* 2007*b*, 2008; Gotsch *et al.* 2010) and to have greater cavitation resistance than low-density woods (Hacke *et al*. 2001; Jacobsen *et al*. 2005; Pratt *et al*. 2007; Lens *et al*. 2011). Wood density has been studied in relation to hydraulic conductivity but the results are inconclusive, showing either a negative relationship between the two traits (measured conductivity, Stratton *et al*. 2000; Bucci *et al*. 2004; Santiago *et al*. 2004) or no relationship (theoretical conductivity, Poorter *et al*. 2010; Fan *et al*. 2012). Also, density has been found to correlate negatively with capacitance (Meinzer *et al.* 2003, 2008; Pratt *et al.* 2007; Scholz *et al.* 2007). Secondly, wood density has been associated with plant mechanical strategies where denser woods tend to be stiffer and more resistant to breakage at a given wood diameter (Chave *et al.* 2009). However, it has also been suggested that plants can build thicker stems to compensate for their lower density (Anten and Schieving 2010; Larjavaara and Muller-Landau 2010; Butler *et al.* 2011). Thirdly, denser woods might be more resistant to pathogen attacks (Augspurger and Kelly 1984; Romero and Bolker 2008). Wood density has also been widely discussed in relation to life-history strategies. For example, species with denser wood tend to experience lower stem mortality rates (Putz *et al.* 1983; Kraft *et al.* 2010; Poorter *et al.* 2010). Growth rate is another important component of life history. Growth rate can be expected to negatively relate to wood density on the basis that higher investment in mass per volume should slow down growth (Enquist *et al.* 1999) and, while generally true, the correlation is not always strong (Poorter *et al.* 2008, 2010; Chave *et al.* 2009; Wright *et al.* 2010; Fan *et al.* 2012). Across species, wood density can vary with environmental factors such as temperature (Wiemann and Williamson 2002; Swenson and Enquist 2007; Martínez-Cabrera *et al.* 2009) and precipitation (Barajas-Morales 1985; Wiemann and Williamson 2002; Swenson and Enquist 2007; Martínez-Cabrera *et al.* 2009; Zhang *et al.* 2011), although not in all studies (Ter Steege and Hammond 2001; Wiemann and Williamson 2002; Muller-Landau 2004). Despite this broad climate-related patterning, wood density also tends to vary quite widely among co-occurring species (Wiemann and Williamson 2002; Muller-Landau 2004).

Thus there are many potential functional roles for wood density but also a number of unresolved questions about each potential role. Larjavaara and Muller-Landau (2010) argued that some observed correlations may not be causal, but rather may reflect correlated selection on other traits. Further, if wood density has multiple functions, then it might not be a very good predictor of any one of them. In any event, a useful step towards resolving this problem is to ask what are the structural underpinnings of wood density variation. The premise of the work reported here was that if structural underpinnings of wood density variation were rigorously quantified and better understood, this might help to explain complexities in the functional implications of wood density.

Most studies of anatomical components of angiosperm wood density span only one or a few species (Schulz 1957; Taylor 1971; Taylor and Wooten 1973; Ezell 1979; Vurdu and Bensend 1980; Fukuzawa 1984; Bosman *et al.* 1994; Stokke and Manwiller 1994; McDonald *et al*. 1995; Lei *et al*. 1996; Denne and Hale 1999; Rana *et al.* 2009) or focus on commercial woods (French 1923 as cited in Panshin and de Zeeuw 1980; Manwiller 1973 as cited in Koch 1985). Fewer studies make comparisons across a broad number of species (Fujiwara *et al.* 1991; Jacobsen *et al.* 2007*a*; Martínez-Cabrera *et al*. 2009; Poorter *et al.* 2010; Fichtler and Worbes 2012).

Wood is a complex tissue composed of three main cell types: vessels that transport water, fibres responsible for mechanical strength, and parenchyma that stores and transports nutrients. These tissues have different structural characteristics and their relative proportions within wood influence wood density. Vessel lumens have essentially zero density; fibre and vessel walls and parenchyma have positive density. Vessel fraction has variously shown either negative or no correlation with wood density (Preston *et al*. 2006; Jacobsen *et al.* 2007*a*; Mitchell *et al*. 2008; Martínez-Cabrera *et al.* 2009; Poorter *et al.* 2010; Zanne *et al.* 2010; Gleason *et al.* 2012). Parenchyma is another commonly occurring tissue, which has been reported to have positive, negative or no relationship with density (Taylor 1969; Fujiwara 1992; Jacobsen *et al.* 2007*a*; Martínez-Cabrera *et al.* 2009; Rana *et al*. 2009; Poorter *et al.* 2010). Wood density is generally well correlated with fibre properties, especially fibre wall fraction (Fujiwara *et al.* 1991; Jacobsen *et al.* 2007*a*; Martínez-Cabrera *et al*. 2009). However, it is unclear how these different fibre traits are interrelated with each other and consequently how these interrelations influence wood density.

Most previous work linking anatomy with density has concentrated on vessels with relatively little attention given to the other tissues. Furthermore, among the studies investigating all the major tissues (vessels, parenchyma and fibres) only one focused on the wood of twigs in 17 species studied by Jacobsen *et al*. (2007*a*). Twigs are important, being in direct spatial and functional contact with leaves and having been commonly subjected to physiological and ecological measurements. In this paper, we investigate wood from twigs from a wide range of angiosperm tree and shrub species growing in various environments (24 species from four sites in eastern Australia). We address two main unresolved issues: (i) Which fibre properties have the most decisive effect on wood density and how are those properties interrelated with each other? (ii) How do vessel and parenchyma proportions influence wood density?

## Methods

### Plant material and sites

Four sites were chosen that spanned a wide range of temperature and aridity in eastern Australia **[see** **Supporting Information****]**. The objective of site selection was to generate a broad range of trait values rather than to enable site comparisons. All carried natural, undisturbed vegetation growing on oligotrophic soils on flat to slightly sloping terrain. Two locations in Tasmania, at ∼43°S, represented low mean annual temperature (MAT; 10.6 °C), and two locations in Queensland near 18°S represented higher MAT (*c*. 22.5 °C). Within each latitude, two locations were chosen so as to differ markedly in aridity index (AI; Willmott and Feddema 1992), the ratio of mean annual precipitation (MAP) to potential evapotranspiration (PET). In both Queensland and Tasmania the wetter site had an AI *c*. 1.0 and the drier site *c*. 0.6. MAP, MAT and PET were obtained from GIS (geographic information system) layers from the Australian Bureau of Meteorology.

At each of the four sites, six abundant and phylogenetically distinct woody eudicot species were chosen for sampling (species listed in Table 1). One species was sampled at two sites, yielding a total of 23 species from eight families. Distal, sun-exposed twigs of trees and shrubs were collected from three replicate individuals per species. The diameter under bark of twigs varied from 4 mm in plants with little pith to 10 mm in plants with higher pith content. Consequently the diameter of wood, excluding bark and pith, was 4–5 mm. This plant material is referred to here as twigs, although in several small shrub species ‘twigs’ were the main stems. Plant material was cut into segments 10–15 cm long and kept wet in sealed plastic bags in the refrigerator (4 °C). Wood density was measured within a week from collection; other parts of the same twigs were placed in fixative for later measurement of anatomical properties (details below).

**Table 1.** Sampled sites, species names and families.

Site	Species name	Family
Cool-wet	*Allocasuarina monilifera*	Casuarinaceae
	*Aotus ericoides*	Fabaceae
	*Banksia marginata*	Proteaceae
	*Eucalyptus amygdalina*	Myrtaceae
	*Leptospermum scoparium*	Myrtaceae
	*Leucopogon ericoides*	Ericaceae
Cool-dry	*Bossiaea cinerea*	Fabaceae
	*Davesia latifolia*	Fabaceae
	*Epacris impressa*	Ericaceae
	*Eucalyptus tenuiramis*	Myrtaceae
	*Leucopogon ericoides*	Ericaceae
	*Persoonia juniperina*	Proteaceae
Hot-wet	*Acacia mangium*	Fabaceae
	*Allocasuarina torulosa*	Casuarinaceae
	*Alphitonia excelsa*	Rhamnaceae
	*Chionanthus ramiflorus*	Oleaceae
	*Eucalyptus platyphylla*	Myrtaceae
	*Ixora timorensis*	Rubiaceae
Hot-dry	*Acacia flavescens*	Fabaceae
	*Corymbia intermedia*	Myrtaceae
	*Gastrolobium grandiflorum*	Fabaceae
	*Grevillea parallela*	Proteaceae
	*Lophostemon suaveolens*	Myrtaceae
	*Persoonia falcata*	Proteaceae

### Wood density

Wood density was measured on segments 3–5 cm long for each twig sample. Bark and pith were removed and measurements were carried out on xylem only. In this paper we refer to xylem as ‘wood’. After removing bark and pith, wood pieces were soaked in water for at least 48 h prior to volume measurement. Then a beaker filled with water was placed on a balance (0.0001 g, Mettler AE 160). A thin wire platform was suspended in water so that it did not touch the side or bottom of the beaker. The balance was tared before each measurement and a sample was gently placed on the platform. The mass of displaced water was read from the balance. From standard water density of 1 g cm^−3^ and knowing the mass of displaced water, we calculated sample volumes applying Archimedes' buoyancy principle (e.g. 1 g of displaced water equals 1 cm^3^ volume). Samples were then placed in paper envelopes and dried at 70 °C for at least 72 h. Wood density was calculated as the dry mass divided by water saturated volume (g cm^−3^).

### Anatomy

To obtain anatomical cross-sections the material was first fixed in formalin–acetic acid–alcohol (FAA) for 4 weeks. The FAA was prepared in proportions of 5 : 5 : 90 (formalin : glacial acetic acid : 70 % ethanol; Gerlach 1972). After 4 weeks, the fixative was replaced with 70 % ethanol. This 70 % ethanol was then replaced two more times within 10 days to further wash the fixative out. Final replacement of alcohol was used as a long-term storage medium. Segments for image analysis were rehydrated by immersion in 50 % ethanol and after 2–5 days in 30 % ethanol. Cross-sections were cut with a sledge microtome (Reichert, Vienna, Austria) at 10–20 µm thickness using disposable blades (Model A35, Feather Safety Razor Co. Ltd, Japan). For better contrast and tissue identification, sections were stained with safranin O (Gurr Microscopy Materials, BDH Chemicals Ltd, UK) for lignified cell walls (10 min) and with Janus green B (Gurr's, London, UK) for cytoplasm (10 min). Safranin O solution used 2 g of stain in 100 mL of distilled water (Ruzin 1999) and Janus green B used 0.1 g of stain and 1 mL of glacial acetic acid in 100 mL of distilled water (Conn *et al.* 1960). Sections were rinsed in distilled water after each staining session. Afterwards, they were mounted in glycerol on a slide, covered with a cover slip and sealed with nail polish. Measurements were made only on cross-sections, but to assist in interpreting and identifying cell types, longitudinal tangential and radial sections and macerations were also made. For macerations, small shavings were placed in vials filled with Franklin's solution: glacial acetic acid and 6 % hydrogen peroxide in proportions of 1 : 1 (Franklin 1945). These vials were loosely covered with Parafilm tape and heated in the oven at 60 °C for 1–2 days. Tissues were then rinsed with distilled water, stained with safranin O (10 min) and gently squashed onto a microscope slide.

**Figure 1.**
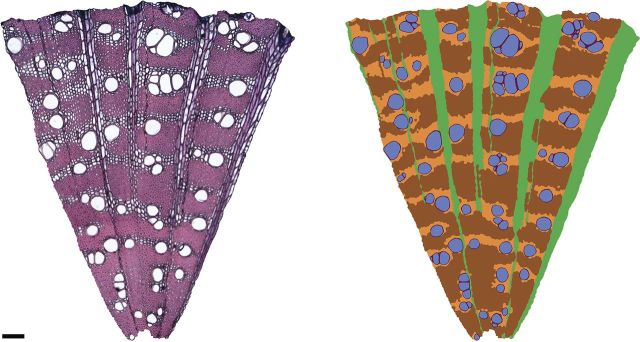
A twig cross-section of *Grevillea parallela*, Proteaceae. The radial sector of the stained section is shown on the left side and the processed image on the right. Colours in the processed image denote different tissue types: blue, vessel lumen; purple, vessel wall; green, rays; orange, axial parenchyma; brown, fibres. The scale bar corresponds to 100 µm.

Microphotographs of cross-sections were taken at ×100 and ×400 magnifications using a digital camera (Scion Corporation, CFW-1310C, USA) attached to a light microscope (Olympus BX 50F, Olympus Co. Ltd, Japan) and image capturing software Scion Visicapture, version 1.4 (Scion Corporation). Two to three images of the same area at different focal planes were taken and stacked in Photoshop CS4 (Adobe Systems Incorporated, USA). Cross-sections were bigger than the field of view; therefore, dozens (for ×100) or around 10 (for ×400) images per cross-section were taken and then merged in Photoshop, giving rise to images of whole cross-sections at ×100 and of one narrow transect at ×400. Tissue cross-sectional areas and vessel traits were measured on one wedge-shaped transect per replicate (×100; Fig. 1) and fibre characteristics (×400) on one rectangular transect, both stretching from pith to cambium. The radial transects were chosen to be the most representative for a section and the tension wood was avoided where possible. The transect borders were approximately parallel to the rays and followed middle lamella so that no open cells were positioned on the borders. Tissue areas, vessels and fibre walls were manually coloured in Photoshop using a Cintiq 21UX graphic tablet (Wacom Co., Ltd, Japan). Protoxylem and newly produced xylem were excluded from analysis. Larger regions were inspected in species with larger vessels or with more variable structure in the tangential direction. The measured area ranged from 0.21 to 0.69 mm^2^ for species from cool sites and from 0.31 to 1.9 mm^2^ for species from hot sites. On average, for each image there were 170 vessels measured (±145) of all sizes including vessel tails. Fibre, fibre wall and lumen areas were measured for an average of 170 fibres per sample (±57) lying in two parallel rows from pith to cambium. Colour-coded images were analysed with Image-Pro Plus version 2.0.0.260 (Media Cybernetics, Inc., USA; Fig. 1).

Proportions of all major wood cell types (vessel wall and lumen, fibre wall and lumen, axial parenchyma, rays and tracheids) and their properties (mean vessel lumen area and mean fibre lumen and wall area) were quantified. Here, mean vessel lumen area is also called ‘mean vessel area’ for brevity. Mean vessel area, fibre lumen area and wall area were calculated as the arithmetic mean across all relevant cells (vessels or fibres) measured within a given radial sector of a sample. In addition to these traits we calculated theoretical maximum hydraulic conductivity (called here ‘theoretical conductivity’; also known as ‘potential conductivity’). Conductivity for the lumen of each conduit was calculated from the Hagen–Poiseuille expression (π*r*^4^)/(8*µ*), where *r* represents mean vessel lumen radius and *µ* represents water dynamic viscosity (assuming a standard viscosity value of 1 at 20 °C). The sum of all vessel lumen conductivities per unit cross-section is theoretical conductivity. This quantity expresses conductivity variation across species due to conduit diameters, but it should be thought of as the *theoretical* maximum since it does not capture effects of end-wall resistances, blockage by tyloses, effects of emboli, etc.

Traditionally, wood is considered a complex tissue composed of several cell types (Evert 2006). However, we refer to those cell types as ‘tissues’ for brevity and also because they perform distinctly different functions. Anatomical terminology follows the ‘IAWA list of microscopic features for hardwood identification’ (IAWA Committee 1989). Vascular and/or vasicentric tracheids occurred in 13 species and are referred to hereafter as tracheids. Tracheids were first determined in macerated wood and then identified on a cross-section on the basis of the number of pits and size of the pit border (both resembling that of vessel pits) and cell size (IAWA Committee 1989; Sano *et al.* 2011). Axial apotracheal and paratracheal parenchyma were collectively measured as axial parenchyma. None of the species studied here had storied rays. Tissue types were expressed as the fraction of a tissue per cross-sectional area (Fig. 1). Tissue fractions of the area outside vessel lumen (non-vessel area) were calculated as tissue fraction multiplied by non-vessel area fraction. The term ‘non-vessel’ area is used for brevity and it includes vessel walls, fibre walls and lumens, axial and ray parenchyma, and tracheids. Non-vessel-lumen quantities are hereafter denoted by subscript ‘NV’, e.g. fibre wall fraction_NV_, wood density_NV_, etc. Fibre wall proportion in a given fibre was expressed as a proportion of the total fibre area. Fibre wall fraction was obtained by multiplying mean fibre wall proportion in a fibre by the fibre fraction per cross-section. Fibre lumen fraction was similarly calculated from mean fibre lumen proportion in a fibre multiplied by fibre fraction.

### Statistical analysis

We collected measurements for 23 species, one of which occurred at two sites and was considered as two entities, giving a total of 24 data points analysed. Measurements were carried out on three replicate individuals per species and the trait values were averaged for comparisons across species. Wood density, vessel lumen, sum of ray and axial parenchyma, ray parenchyma and fibre wall fractions of total wood area as well as non-vessel wood area were all approximately normally distributed (Shapiro–Wilk test, *P* < 0.05). Vessel wall, axial parenchyma, tracheids and fibre lumen fractions of total wood area as well as of non-vessel wood area were right-skewed and were transformed to generate approximately normally distributed variables. Log_10_ transformations normalized all distributions with the exception of tracheid fraction and tracheid non-vessel fraction. We used ordinary least-squares regression to assess bivariate relationships (SigmaPlot, Systat, San Jose, CA, USA).

## Results

**Figure 2.**
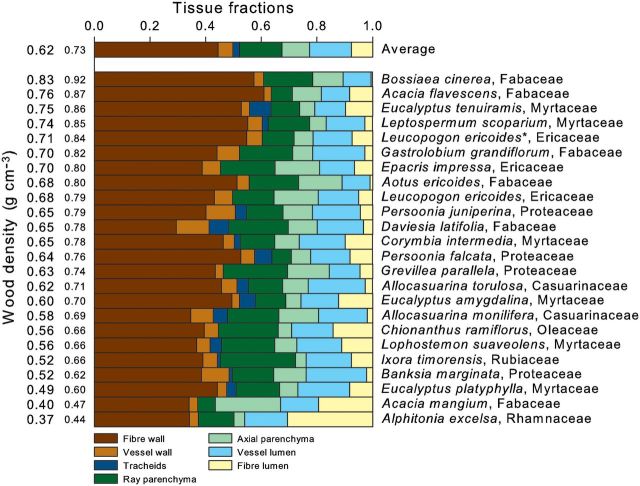
Tissue fractions for 24 species arranged in order of increasing wood density (from bottom to top). Large wood density numbers indicate total wood density whereas small numbers indicate non-vessel density (density_NV_). Mean tissue fractions across all species are shown in the bar at the top of the figure. *Leucopogon ericoides* occurred in two sites and is treated here as two separate entities. An asterisk denotes *L. ericoides* from the cold-wet site as opposed to *L. ericoides* from the cold-dry site with no indicator.

Wood density and tissue proportions varied significantly across species as illustrated in Fig. 2 **[see** **Supporting Information****]**. Wood density varied more than 2-fold, from 0.37 to 0.83 g cm^−3^. Figure 2 shows tissue fractions averaged across all species (bar at the top) and for each individual species separately (the remaining bars). The mean fibre fraction was 0.52 ± 0.09 (hereafter numbers represent average fraction ± one standard deviation). Fibre varied approximately 2-fold across species and was the most abundant tissue type. Fibre fraction could be partitioned into fibre walls (0.45 ± 0.08, 2-fold variation; brown bars in Fig. 2) and fibre lumens (0.08 ± 0.07, *c.* 60-fold variation; yellow bars). On average, parenchyma occupied 0.25 of wood cross-sectional area and varied almost 3-fold across species. It consisted of axial parenchyma (0.10 ± 0.05, 6-fold variation) and ray parenchyma (0.15 ± 0.05, 4.5-fold variation). Vessels occupied 0.20, where 0.15 ± 0.03 consisted of lumens (varying *c.* 2-fold) and 0.05 ± 0.03 of vessel walls (varying 4.5-fold). Tracheids occurred in just 13 of 24 species and occupied only small fractions (0.02 ± 0.03 averaged across all 24 species, 8.5-fold variation across the 13 species that had tracheids).

**Figure 3.**
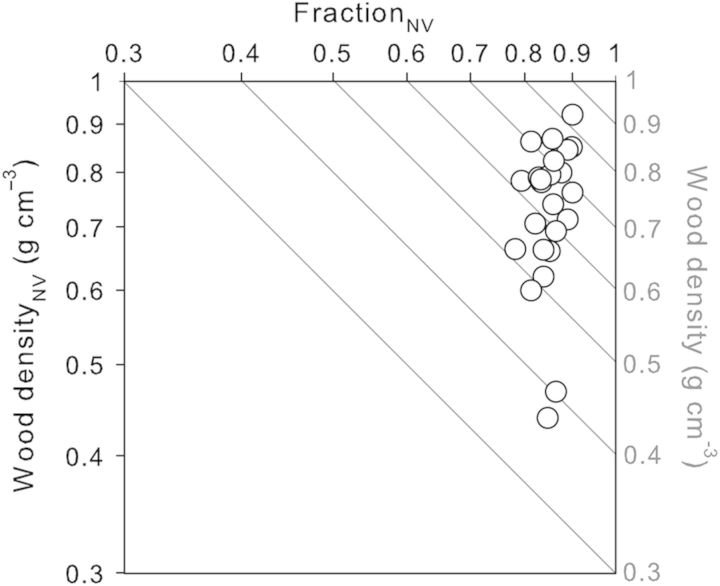
Relationship between fraction of wood outside vessel lumens (fraction_NV_) and the density of that non-vessel fraction (wood density_NV_) among 24 Australian species. Fraction_NV_ = 1−vessel fraction. Each circle represents a different species (mean value from three replicates). Diagonal isolines represent contours of overall wood density, which increases towards the upper right. All axes are log scaled.

Alternatively, wood can simply be divided into two components: vessel lumen fraction and non-vessel fraction, which encompasses all tissues other than vessel lumens. The density of the non-vessel fraction and tissue fractions within the non-vessel fraction are indicated hereafter by the subscript ‘NV’, e.g. density_NV_, fibre fraction_NV_. Since vessel lumen has zero density, overall wood density is (by definition) the product of the non-vessel fraction density (density_NV_) and the non-vessel fraction itself (fraction_NV_): density = density_NV_ × fraction_NV_ (Preston *et al.* 2006; Zanne *et al.* 2010). These three quantities can be log transformed and the equation then becomes a sum: log(density) = log(density_NV_) + log(fraction_NV_). Hence, when density_NV_ is plotted against fraction_NV_ on log–log axes (Fig. 3), isolines of resulting overall wood density can be constructed and used to aid interpretation. Variation across species in density_NV_ (*y*-axis in Fig. 3) was four times greater than variation in fraction_NV_ (*x*-axis), and thus in this species set was a far stronger determinant of variation in overall wood density (direction across the isolines). Not surprisingly then, density_NV_ and overall density were tightly correlated with each other (*r*^2^ = 0.95, *P* < 0.001). Vessel lumen fraction was only loosely (negatively) correlated with overall wood density (*r*^2^ = 0.20, *P* = 0.027). Therefore, the following analyses concentrate entirely on density_NV_ and its anatomical components **[see** **Supporting Information****]**.

**Figure 4.**
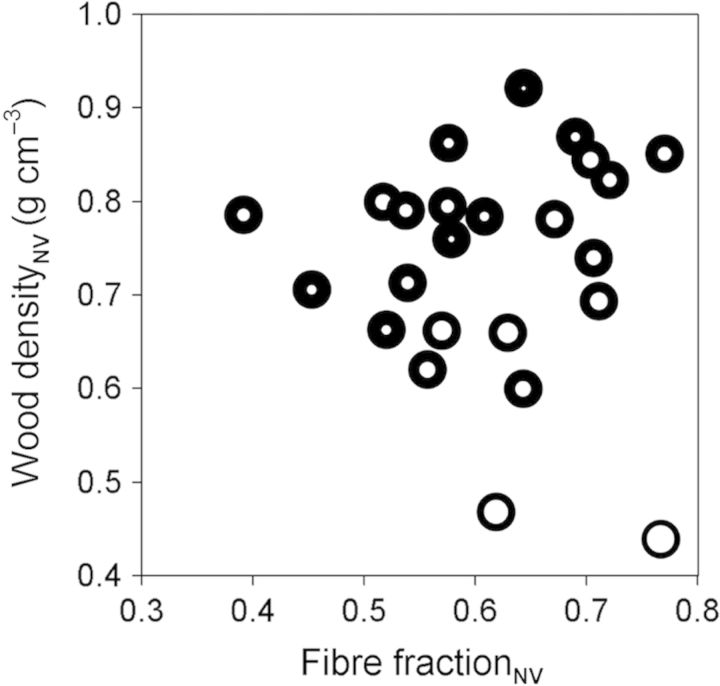
Relationship between non-vessel density (wood density_NV_) and fibre fraction in non-vessel fraction (fibre fraction_NV_). Each ‘donut’ circle symbolizes one species. The width of the donut border (black) represents fibre wall proportion within an individual fibre and the width of the hole (white) represents fibre lumen proportion. These proportions were estimated from individual fibres (as fibre wall area—or lumen area—divided by total fibre area), for 75–314 fibres per replicate (mean 170), and then across three replicates per species.

Total fibre fraction_NV_ (fibre lumen fraction_NV_ plus fibre wall fractions_NV_) was unrelated to density_NV_ (Fig. 4). Species with the same fibre fraction_NV_ varied widely in wall proportion relative to lumen within a fibre (as indicated by the width of the ‘donut’ rings in Fig. 4). Figure 4 shows that lowest-density (<0.5 g cm^−3^) species had high fibre fraction_NV_ but their fibres had low wall proportion (lower right of the graph). High-density_NV_ species (>0.85 g cm^−3^) also had high fibre fraction_NV_ but their fibres had large wall proportion (upper right of the graph). A substantial number of species located in the middle of the graph with medium density_NV_ (0.60–0.85 g cm^−3^) had variable fibre fraction_NV_ and fibre wall proportion within a fibre. Also, fibre wall proportion within a fibre was positively correlated with wood density_NV_ (*r*^2^ = 0.62, *P* < 0.001).

**Figure 5.**
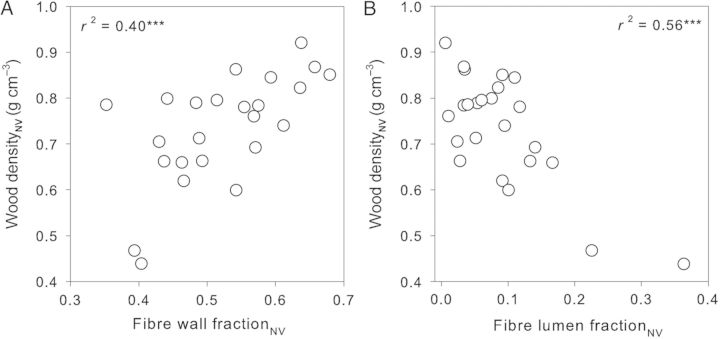
Relationships between non-vessel density (wood density_NV_) and (A) fibre wall fraction in non-vessel fraction (fibre wall fraction_NV_) and (B) fibre lumen fraction in non-vessel fraction (fibre lumen fraction_NV_). Each circle represents a different species (mean value from three replicates). ***P < 0.001.

Density_NV_ was positively correlated with fibre wall fraction_NV_ (*r*^2^ = 0.40; Fig. 5A), and negatively with fibre lumen fraction_NV_ (*r*^2^ = 0.56; Fig. 5B). Since the majority of species had low fibre lumen fraction_NV_ (i.e. the data were right-skewed), the two species with highest fibre lumen fraction_NV_ had a strong influence on these relationships (lower left of Fig. 5A, lower right of Fig. 5B). That said, the correlations were still present across the other species considered on their own (*r*^2^ = 0.26 and *P* = 0.014, *r*^2^ = 0.18 and *P* = 0.049, respectively). The species with the lowest fibre wall fraction_NV_ (upper left in Fig. 5A) was *Daviesia latifolia*, which had a high amount (fraction of 0.07) of thick-wall tracheids. Presumably, this high fraction of tracheid wall contributed to the quite high density_NV_ of this species (high, given its very low fibre fraction).

**Figure 6.**
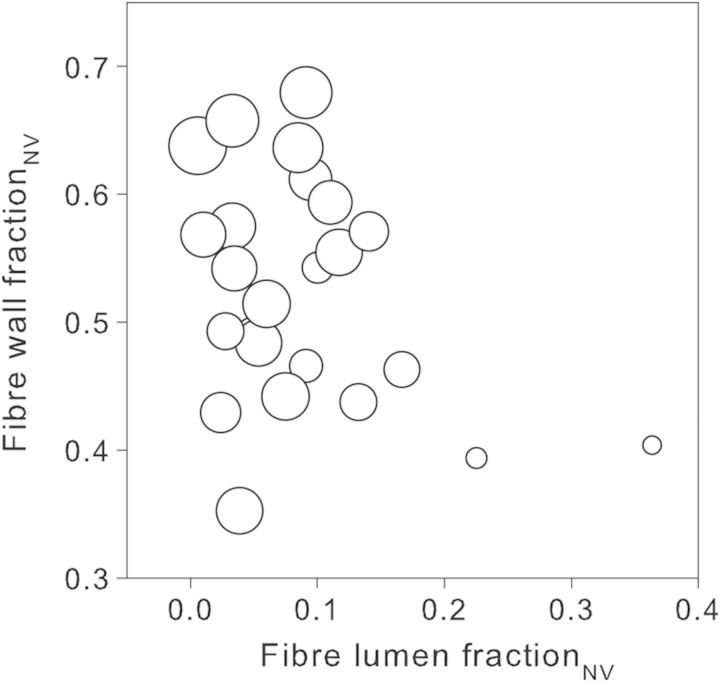
Relationship between the fraction of the non-vessel fraction that is fibre wall or is fibre lumen. Each symbol represents a different species (mean value from three replicates). Symbol diameter is proportional to non-vessel wood density (wood density_NV_), with the biggest symbol indicating highest density_NV_ (0.92 g cm^−3^) and the smallest symbol indicating lowest density_NV_ (0.44 g cm^−3^).

Next we asked how fibre wall and lumen fractions_NV_ were related to one another. Figure 6 shows an approximately triangular relationship between fibre wall fraction_NV_ and fibre lumen fraction_NV_. The highest-density_NV_ species (>0.85 g cm^−3^; four large symbols, upper left of Fig. 6) had high fibre wall fraction_NV_ and low fibre lumen fraction_NV_. Medium-density_NV_ species (0.60–0.85 g cm^−3^; 18 medium-sized symbols in Fig. 6) had variable fibre wall fraction_NV_ and variable fibre lumen fraction_NV_. The two lowest-density_NV_ (<0.50 g cm^−3^) species had the highest fibre lumen fraction_NV_ and low fibre wall fraction_NV_ (two smallest symbols, lower right of Fig. 6). The categories of high-, medium- and low-density species are used here for easy reference, but in fact, the trait values are continuous and no clear boundaries can be indicated. The species with lowest fibre wall fraction_NV_ (lower left in Fig. 6) was *D. latifolia* (see the comment about tracheids above).

The second most abundant tissue, parenchyma, was not correlated with density_NV_ nor were its components, rays and axial parenchyma (all *P*> 0.7). Similarly, vessel wall fraction_NV_ was unrelated to density_NV_. Nevertheless, both parenchyma and vessel wall fractions_NV_ indirectly affected density_NV_. Figure 6 illustrates that there was considerable variation in density_NV_ (indicated by symbol size) at a given fibre wall fraction_NV_, especially at lower wall fraction_NV_ (lower half of the graph), and considerable variation in density_NV_ at a given fibre lumen fraction_NV_, especially at lower lumen fraction_NV_ (left half of the graph). These variations in density_NV_ could be partially explained by parenchyma and vessel wall fractions_NV_. At a given fibre wall fraction_NV_, density_NV_ was positively correlated with parenchyma and vessel wall fractions_NV_ (*r*^2^ = 0.15, *P* = 0.064 and *r*^2^ = 0.29, *P* = 0.007, respectively) and negatively with fibre lumen fraction_NV_ (*r*^2^ = 0.45, *P* < 0.001; all relationships tested against residuals from a regression of wood density_NV_ on fibre wall fraction_NV_). Conversely at a given fibre lumen fraction_NV_ (i.e. tested against residuals from a regression of wood density_NV_ on fibre lumen fraction_NV_), wood density_NV_ was negatively correlated with parenchyma fraction_NV_ (*r*^2^ = 0.28, *P* = 0.008), not correlated with vessel wall fraction_NV_ (*P* = 0.29) and positively correlated with fibre wall fraction_NV_ (*r*^2^ = 0.40, *P* < 0.001). Additionally, parenchyma fraction_NV_ was tightly negatively correlated with total fibre fraction_NV_ (*r*^2^ = 0.74, *P* < 0.001). The only remaining tissue, tracheids, occupied on average a very small fraction_NV_ and was not subjected to detailed analysis.

**Figure 7.**
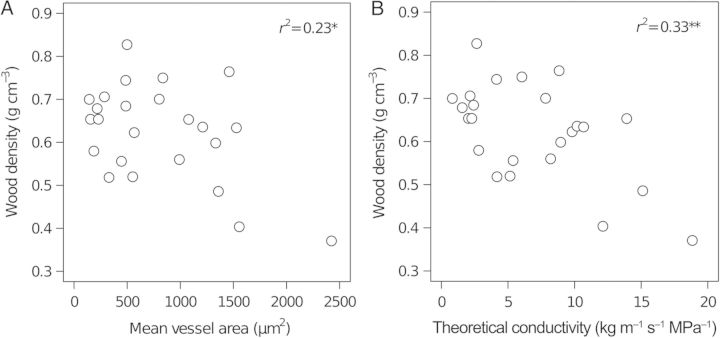
Relationships between wood density and (A) mean vessel area and (B) theoretical conductivity. Each circle represents a different species (mean value from three replicates). *P < 0.05, **P < 0.01.

Mean vessel area and theoretical conductivity **[see** **Supporting Information****]** were negatively correlated with overall wood density across the species studied (*r*^2^ = 0.225, *P* = 0.019 and *r*^2^ = 0.33, *P* = 0.0036, respectively, Fig. 7A and B). However, these relationships were mainly driven by a few species with particularly large vessel lumens and theoretical conductivity (Fig. 7A and B). Among the majority of species there was considerable variation in mean vessel area and conductivity at a given wood density, and little relationship between the two (Fig. 7A and B).

## Discussion

This study aimed to describe the anatomical components of wood density in twigs of 24 Australian tree and shrub species. Properties of fibres, the most abundant tissue, had the strongest effect on wood density variation, as has been shown in some previous studies (Fujiwara *et al.* 1991; Jacobsen *et al.* 2007*a*; Martínez-Cabrera *et al*. 2009). However, our results contribute to a more comprehensive understanding of wood structure and its influence on density in twigs. Here, we discuss in detail the properties of fibres and other tissues as components of density.

### Wood density and its anatomical components

Density of tissue outside vessel lumens (density_NV_), rather than vessel lumen fraction, was the main driver of overall wood density variation. This result agrees with a comparison made across 584 species that considered main stem wood (Zanne *et al*. 2010), implying that density_NV_ determines overall density in both twigs (this study) and main stems similarly. Accordingly, the discussion here is directed towards density_NV_ and individual tissue fractions within the non-vessel part of the wood (indicated by subscript ‘NV’, e.g. fibre wall fraction_NV_). We compare these results with the results for overall wood density reported by other studies, on the basis that overall wood density and density_NV_ are closely correlated (*r*^2^ = 0.95, *P* < 0.001, this study).

All tissue fractions_NV_ influence density_NV_ but fibre wall and lumen fractions_NV_ are the most important. The strong influence of fibre wall fraction_NV_ was due both to its high proportion and its variability, while the influence of fibre lumen fraction_NV_ was associated chiefly with its high variability (*c*. 60-fold). Other studies have consistently found a positive relationship between fibre wall fraction and density: in trunk wood among 50 Japanese trees (Fujiwara *et al.* 1991) and 61 North and South American shrubs (Martínez-Cabrera *et al.* 2009); and in twig wood of 17 South African shrubs (Jacobsen *et al.* 2007*a*). Fibre lumen fraction has received less attention but has also been shown (in concordance with our study) to have a negative relationship with wood density (Martínez-Cabrera *et al.* 2009; Rana *et al.* 2009). The second most abundant tissue, parenchyma, did not correlate with density_NV_ in our study nor in tree and shrub trunks (Martínez-Cabrera *et al.* 2009; Poorter *et al.* 2010; Fichtler and Worbes 2012) but correlated negatively with twig wood density across 17 species (Jacobsen *et al.* 2007*a*). These discrepancies might be caused by variable densities of parenchyma tissue itself (Taylor 1969; Fujiwara 1992; Guilley and Nepveu 2003) or by varied correlations between parenchyma and fibre wall and lumen fractions (see below). Plausibly, these discrepancies could also stem from different relationships between ray and axial parenchyma. We did not find any cross-correlation between those two components of parenchyma nor were they individually related to density. In contrast, Martínez-Cabrera *et al.* (2009) reported that ray and axial parenchyma were negatively correlated with each other and individually correlated with density (axial parenchyma positively correlated, rays—negatively). In that study these links were strongly associated with environmental variables (MAP, MAT and AI). These findings imply that different functional trade-offs can be related to ray and axial parenchyma individually and also that their link is affected by climate. Possibly these trade-offs may be more pronounced in trunk wood, as opposed to the twig wood studied here.

We did not find a direct relationship between parenchyma and density_NV_. Nevertheless, our results imply that parenchyma together with fibre lumens can influence density_NV_ variation in a less direct way. At a given fibre wall fraction_NV_, density_NV_ depended on the parenchyma fraction_NV_ relative to fibre lumen fraction_NV_. Density_NV_ was marginally positively correlated with parenchyma fraction_NV_ and negatively with fibre lumen fraction_NV_. Parenchyma has higher density than fibre lumen, which has zero density. Therefore, higher parenchyma fraction_NV_ relative to fibre lumen fraction_NV_ increases density_NV_, and conversely higher fibre lumen fraction_NV_ decreases density_NV_. We note that parenchyma fraction_NV_ was only weakly correlated with density_NV_ at a given fibre wall fraction_NV_. This weak correlation possibly stems from variable parenchyma tissue densities (Taylor 1969; Fujiwara 1992; Guilley and Nepveu 2003).

Fibre fraction_NV_ (sum of fibre wall and lumen fractions_NV_) was not associated with density_NV_ because of wide variation in wall proportion within a fibre (top right to low right in Fig. 4). Poorter *et al.* (2010) suggested a similar explanation, but as far as we are aware, this issue has not been quantitatively clarified until now.

Anatomical traits that are not expressed as fractions of wood volume have less direct effects on density. We found that density_NV_ was correlated with fibre wall proportion within a fibre. Previous studies have also shown that density can be related to fibre lumen diameter (Jacobsen *et al.* 2007*a*; Martínez-Cabrera *et al*. 2009), fibre wall thickness (Fujiwara *et al.* 1991; Martínez-Cabrera *et al.* 2009) and fibre wall to lumen ratio (Martínez-Cabrera *et al.* 2009). We believe those traits would deliver a more insightful understanding of wood density when combined with information about fractions, e.g. the relationship among density_NV_, fibre fraction_NV_ and fibre wall proportion in a fibre, as described above. Similarly for mean vessel area, which in this study was weakly negatively correlated with wood density. Mean vessel area *per se* should not affect wood density. Rather, vessel fraction (vessel area multiplied by vessel number per area) should be causally linked with density variation.

### Variability of anatomical structures

**Figure 8.**
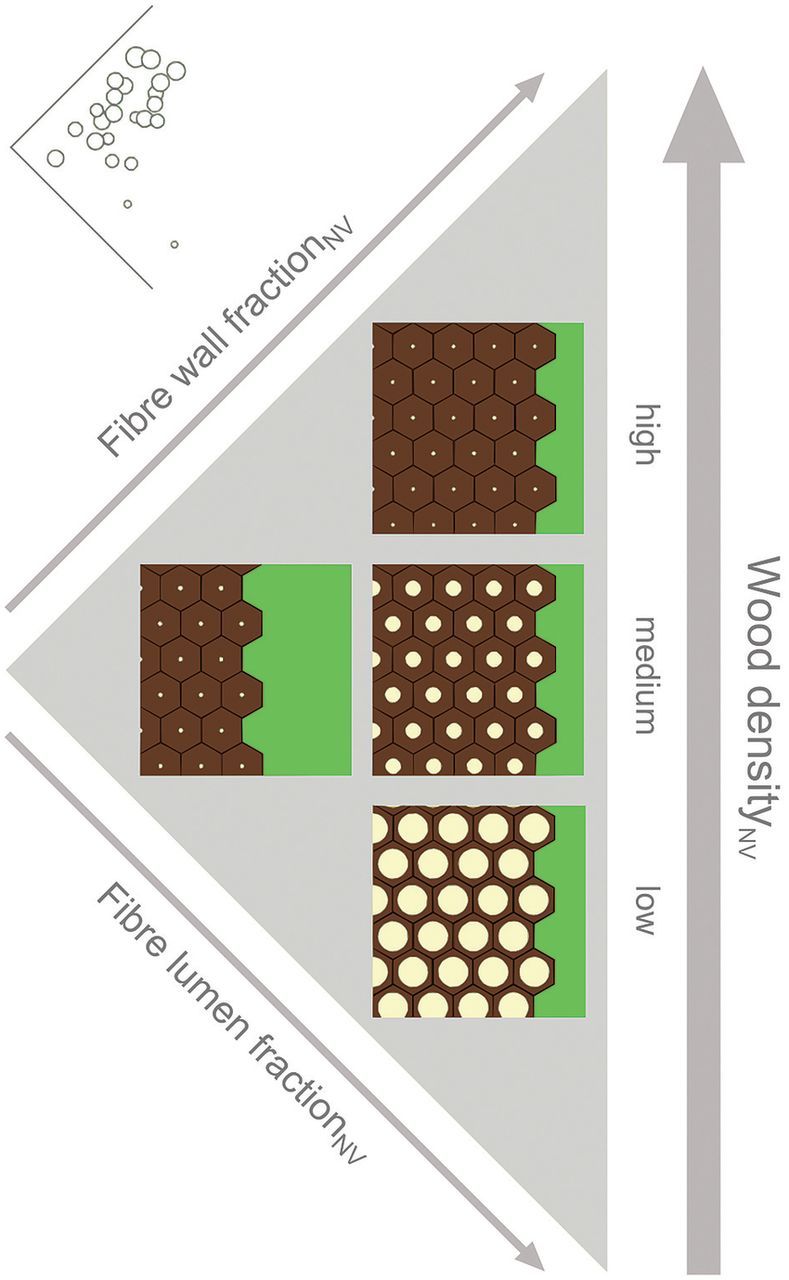
A schematic representation of the graph in Fig. 6 flipped clockwise by 45°. The actual graph from Fig. 6 is repeated here in an inset and also flipped 45° clockwise. The diagram represents four cross-sections of potential anatomical structures in low-, medium- and high-density species. Each hexagon within the three squares indicates a fibre cell consisting of dark fibre wall and bright fibre lumen. The green area on the right of each square indicates parenchyma. Wood density_NV_ increases towards the top of the diagram.

**Figure 9.**
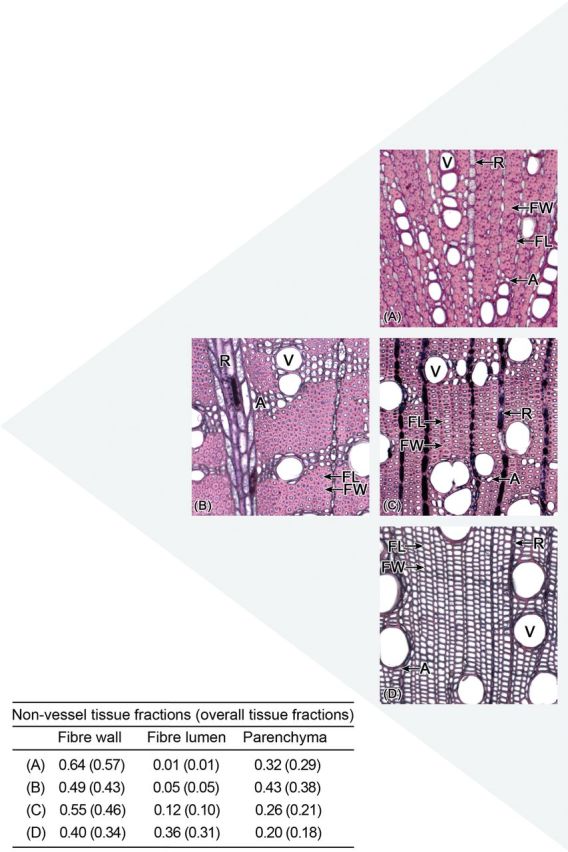
Cross-sections through twigs of four species. The triangle arrangement corresponds to the one in Fig. 8 and so the cross-sections are examples of respective anatomies drawn in that figure. (A) *Bossiaea cinerea* (Fabaceae, wood density_NV_ 0.92 g cm^−3^), (B) *Grevillea parallela* (Proteaceae, wood density_NV_ 0.74 g cm^−3^), (C) *Corymbia intermedia* (Myrtaceae, wood density_NV_ 0.78 g cm^−3^), (D) *Alphitonia excelsa* (Rhamnaceae, wood density_NV_ 0.44 g cm^−3^). V, vessel; FW, fibre wall; FL, fibre lumen; A, axial parenchyma; R, ray parenchyma. Fibre lumens are indistinct in (A) due to their small sizes.

The discussion so far has focused on wood density variation. However, we also found considerable anatomical variation within a given range of density. Hereafter, we use the arbitrary categories of ‘medium’, ‘high’ and ‘low’ density and tissue fractions. However, we observed a continuum of trait values and the categories are used only for convenience. Species with medium density_NV_ (0.60–0.85 g cm^−3^) showed broader structural variability than high- and low-density_NV_ species (>0.85 and <0.5 g cm^−3^, respectively). The concept is illustrated in Fig. 8 and examples of cross-sections approximately corresponding to Fig. 8 are shown in Fig. 9. Species with high density_NV_ (large symbols in Fig. 6, top corner in Figs 8 and 9A) had the highest fraction_NV_ of fibre wall and small fibre lumen fraction_NV_. Their total fibre fraction_NV_ was high and parenchyma fraction_NV_ was low. In contrast, medium-density_NV_ species had more variable fibre wall, fibre lumen and parenchyma fractions_NV_ (medium symbols in Fig. 6, middle in Figs 8 and 9B and C). Consequently, a spectrum of possible architectures may be outlined where the two ends of the spectrum are (i) low fibre, low fibre lumen and high parenchyma fractions_NV_ (middle left in Figs 8 and 9B) and (ii) high fibre, medium fibre lumen and low parenchyma fractions_NV_ (middle right in Figs 8 and 9C). The lowest-density species (<0.5 g cm^−3^, small symbols in Fig. 6, bottom corner in Figs 8 and 9D) in this study had high fibre lumen fraction_NV_. However, it is possible that low-density wood could also be composed of large parenchyma fractions_NV_ and small fibre lumen fractions_NV_. More studies are needed to clarify the range of anatomies exhibited by low-density species. To our knowledge, we are the first to set out this triangular scheme relating wood anatomy to density. Variability of structures in medium-density species implies that there may be a wider range of ecological strategies available to these species.

### Ecological and physiological considerations

It has been shown that denser woods tend to operate at more negative minimum water potentials (Ackerly 2004; Bucci *et al.* 2004; Santiago *et al.* 2004; Jacobsen *et al.* 2007*b*, 2008; Gotsch *et al.* 2010) and have higher cavitation resistance (Hacke *et al*. 2001; Jacobsen *et al*. 2005; Pratt *et al*. 2007; Lens *et al*. 2011). However, we and previous literature have shown that wood density is most strongly related to fibre properties, not vessel properties. Thus, the relationship among wood density, minimum water potential and cavitation resistance does not appear to be directly influenced by wood density, but rather by a third, unexplained factor. Another element of hydraulic strategies is capacitance, which tends to be negatively correlated with wood density (Meinzer *et al.* 2003, 2008; Pratt *et al.* 2007; Scholz *et al.* 2007). Our results imply that capacitance water could be stored in fibre lumen or in parenchyma but the exact mechanism of this water storage and release is not known, nor is it clear what might be the advantage or disadvantage of fibre lumen capacitance compared with parenchyma capacitance. Density also plays a role in the mechanical behaviour of wood. It is a good predictor of mechanical strength and elasticity at a given wood diameter (Chave *et al.* 2009). However, it has also been shown that plants can compensate for mechanically weak wood by building thicker stems (Anten and Schieving 2010; Larjavaara and Muller-Landau 2010; Butler *et al.* 2011). Thick, low-density stems could be mechanically strong but their maintenance costs would be higher due to large surface area (Larjavaara and Muller-Landau 2010). Additionally, our results suggest that in similar density woods maintenance costs per sapwood cross-sectional area could vary depending on wood anatomical structure. Namely, woods with high fraction of living parenchyma would presumably incur higher maintenance costs than woods with high fraction of non-living fibres. What then would be the advantage of high parenchyma fraction? Hypothetically, high parenchyma fraction could ensure high nutrient storage capacity, which may be beneficial in some ecological strategies. This issue and other questions about causal links between wood anatomical structure and ecological strategies require further investigation and open interesting pathways for future research.

### Twig and main trunk comparisons

Caution is needed when comparing results from twigs with results from main trunks. It is not proven that the relationships of wood density and anatomy are the same at the twig level as at the trunk level. Taylor and Wooten (1973) found that in five species vessel and fibre fractions shifted in the same direction with plant height, but this was not the case for ray fraction. Other studies examining association between vessel lumen fraction and wood density across a large number of species showed no relationship in the main trunks (Martínez-Cabrera *et al.* 2009; Poorter *et al.* 2010; Zanne *et al.* 2010) but a negative relationship in twigs (Preston *et al.* 2006; Jacobsen *et al.* 2007*a*; Mitchell *et al*. 2008; Gleason *et al.* 2012). Such disparities indicate that the relationship between wood density and tissue fractions may conceivably be different in main trunks than in twigs, yet it is unclear how generally this is so.

## Conclusions

Wood density has been proposed as a key plant functional trait and is related to ecological strategies (Chave *et al.* 2009) but relatively little is known about the underpinnings of these relationships. This study clarifies the anatomical basis for wood density variation across species. It shows that wood density depends on anatomical structure, but also that a range of very different structures can result in very similar wood density, especially among species with medium density_NV_ (here, 0.60–0.85 g cm^−3^). This conclusion suggests that there may be a wider range of ecological strategies among such species. Taken together, these findings imply that twig wood density should not be considered as an unambiguous indicator of plant ecological strategies. We hope this research will enhance the interpretation and design of ecological studies related to wood density.

## Sources of Funding

This work has been supported by the Macquarie University Research Excellence Scholarship (Australia) and the Australian Research Council Discovery Project grant DP0877064 awarded, respectively, to K.Z. and M.W.

## Contributions by the Authors

All authors contributed to the analysis of the results, writing and editing of the manuscript. D.W.B devised site selection. K.Z., D.W.B and S.M.G. carried out fieldwork. K.Z. performed anatomical work.

## Conflicts of Interest Statement

None declared.

## Supporting Information

The following Supporting Information is available in the online version of this article –

**File 1.** Table. Details of the four sites sampled in this study.

**File 2.** Table. Species traits: wood density and tissue fractions.

**File 3.** Table. Species traits: wood density and tissue fractions of non-vessel fraction.

**File 4.** Table. Species traits: mean vessel area and theoretical conductivity.

Additional Information
