# Supplementary material for: Fibre wall and lumen fractions drive wood density variation across 24 Australian angiosperms
Source: AoB Plants. 2013 Oct 10;5:plt046. doi: 10.1093/aobpla/plt046 (PMC4104653; doi:10.1093/aobpla/plt046)
Supplement: Additional Information [file supp_5_plt046_index.html]

Fibre wall and lumen fractions drive wood density variation across 24 Australian angiosperms — Fibre wall and lumen fractions drive wood density variation across 24 Australian angiosperms — Additional Information 

# Fibre wall and lumen fractions drive wood density variation across 24 Australian angiosperms

## Additional Information

Additional Information

**Files in this Data Supplement:**

- Additional Information Table 1 - docx file
- Additional Information Table 2 - docx file
- Additional Information Table 3 - docx file
- Additional Information Table 4 - docx file
