# Supplementary material for: Fibre wall and lumen fractions drive wood density variation across 24 Australian angiosperms
Source: AoB Plants. 2013 Oct 10;5:plt046. doi: 10.1093/aobpla/plt046 (PMC4104653; doi:10.1093/aobpla/plt046)
Supplement: Additional Information [file supp_plt046_plt046supp_table1.docx]

Details of the four sites sampled in this study.

|  | Hot-wet  Cardwell (QLD) | Hot-dry  Princess Hills (QLD) | Cool-wet  Lower Longley (TAS) | Cool-dry  Bothwell (TAS) |
| --- | --- | --- | --- | --- |
|  |  |  |  |  |
| Sampling time | August 2009 | November 2009 | March 2009 | March 2009 |
| Biome | tropical dry forest | savanna | woodland | woodland |
| Longitude | 146.16°S | 145.52°S | 147.18°S | 147.04°S |
| Latitude | 18.48°E | 18.25°E | 42.98°E | 42.39°E |
| Altitude (m) | 50 | 595 | 280 | 420 |
| MAP(mm) | 1925 | 1106 | 964 | 547 |
| MAT(°C) | 24.1 | 21.3 | 11.3 | 10 |
| AI(MAP/PET) | 1.026 | 0.601 | 1.153 | 0.646 |

MAP – mean annual precipitation, MAT – mean annual temperature, AI – aridity index, QLD – Queensland, TAS – Tasmania
