# Supplementary material for: Fibre wall and lumen fractions drive wood density variation across 24 Australian angiosperms
Source: AoB Plants. 2013 Oct 10;5:plt046. doi: 10.1093/aobpla/plt046 (PMC4104653; doi:10.1093/aobpla/plt046)
Supplement: Additional Information [file supp_plt046_plt046supp_table2.docx]

Wood density and tissue fractions of 24 species averaged across three replicates^[[1]](#footnote-1)^*.

| Site | Species | Wood density (g cm^-3^) | Fibre | Fibre wall | Fibre lumen | Parenchyma | Axial parenchyma | Ray  parenchyma | Vessel lumen | Vessel wall | Tracheids | Fibre wall proportion in a fibre | Fibre lumen proportion in a fibre |
| --- | --- | --- | --- | --- | --- | --- | --- | --- | --- | --- | --- | --- | --- |
| Cool-wet | *Allocasuarina monilifera* | 0.58 | 0.37 | 0.35 | 0.02 | 0.33 | 0.15 | 0.19 | 0.18 | 0.08 | 0.05 | 0.95 | 0.05 |
|  | *Aotus ericoides* | 0.68 | 0.52 | 0.51 | 0.01 | 0.33 | 0.16 | 0.18 | 0.10 | 0.04 | na | 0.98 | 0.02 |
|  | *Banksia marginata* | 0.52 | 0.41 | 0.39 | 0.02 | 0.27 | 0.12 | 0.15 | 0.22 | 0.10 | 0.01 | 0.95 | 0.05 |
|  | *Eucalyptus amygdalina* | 0.60 | 0.62 | 0.49 | 0.12 | 0.16 | 0.05 | 0.11 | 0.14 | 0.03 | 0.06 | 0.80 | 0.20 |
|  | *Leptospermum scoparium* | 0.74 | 0.59 | 0.56 | 0.03 | 0.20 | 0.06 | 0.15 | 0.14 | 0.05 | 0.02 | 0.95 | 0.05 |
|  | *Leucopogon ericoides* | 0.71 | 0.62 | 0.55 | 0.07 | 0.18 | 0.07 | 0.11 | 0.14 | 0.06 | na | 0.88 | 0.12 |
| Cool-dry | *Bossiaea cinerea* | 0.83 | 0.58 | 0.57 | 0.01 | 0.29 | 0.11 | 0.18 | 0.10 | 0.03 | na | 0.99 | 0.01 |
|  | *Davesia latifolia* | 0.65 | 0.33 | 0.29 | 0.03 | 0.32 | 0.10 | 0.21 | 0.17 | 0.12 | 0.07 | 0.91 | 0.09 |
|  | *Epacris impressa* | 0.70 | 0.45 | 0.39 | 0.07 | 0.36 | 0.16 | 0.20 | 0.12 | 0.07 | na | 0.85 | 0.15 |
|  | *Eucalyptus tenuiramis* | 0.75 | 0.63 | 0.53 | 0.10 | 0.16 | 0.06 | 0.10 | 0.11 | 0.03 | 0.08 | 0.84 | 0.16 |
|  | *Leucopogon ericoides* | 0.68 | 0.49 | 0.44 | 0.05 | 0.31 | 0.16 | 0.15 | 0.15 | 0.07 | na | 0.90 | 0.10 |
|  | *Persoonia juniperina* | 0.65 | 0.45 | 0.40 | 0.04 | 0.24 | 0.11 | 0.13 | 0.17 | 0.11 | 0.04 | 0.90 | 0.10 |
| Hot-wet | *Acacia mangium* | 0.40 | 0.54 | 0.34 | 0.19 | 0.30 | 0.23 | 0.06 | 0.14 | 0.03 | na | 0.64 | 0.36 |
|  | *Allocasuarina torulosa* | 0.62 | 0.48 | 0.46 | 0.03 | 0.21 | 0.09 | 0.12 | 0.21 | 0.06 | 0.04 | 0.95 | 0.05 |
|  | *Alphitonia excelsa* | 0.37 | 0.65 | 0.34 | 0.31 | 0.17 | 0.04 | 0.13 | 0.15 | 0.03 | na | 0.53 | 0.47 |
|  | *Chionanthus ramiflorus* | 0.56 | 0.54 | 0.40 | 0.14 | 0.26 | 0.05 | 0.21 | 0.15 | 0.05 | na | 0.74 | 0.26 |
|  | *Eucalyptus platyphylla* | 0.49 | 0.52 | 0.44 | 0.08 | 0.22 | 0.07 | 0.16 | 0.19 | 0.03 | 0.03 | 0.85 | 0.15 |
|  | *Ixora timorensis* | 0.52 | 0.47 | 0.39 | 0.08 | 0.31 | 0.04 | 0.27 | 0.16 | 0.05 | 0.01 | 0.84 | 0.16 |
| Hot-dry | *Acacia flavescens* | 0.76 | 0.69 | 0.61 | 0.08 | 0.18 | 0.10 | 0.07 | 0.10 | 0.03 | na | 0.88 | 0.12 |
|  | *Corymbia intermedia* | 0.65 | 0.56 | 0.46 | 0.10 | 0.21 | 0.09 | 0.12 | 0.17 | 0.04 | 0.02 | 0.83 | 0.17 |
|  | *Gastrolobium grandiflorum* | 0.70 | 0.47 | 0.44 | 0.03 | 0.26 | 0.07 | 0.19 | 0.19 | 0.08 | na | 0.94 | 0.06 |
|  | *Grevillea parallela* | 0.63 | 0.48 | 0.43 | 0.05 | 0.38 | 0.15 | 0.23 | 0.11 | 0.03 | na | 0.90 | 0.10 |
|  | *Lophostemon suaveolens* | 0.56 | 0.48 | 0.37 | 0.11 | 0.27 | 0.08 | 0.19 | 0.16 | 0.05 | 0.04 | 0.76 | 0.24 |
|  | *Persoonia falcata* | 0.64 | 0.61 | 0.53 | 0.08 | 0.14 | 0.07 | 0.07 | 0.14 | 0.05 | 0.06 | 0.86 | 0.14 |

1. * Notes: all values, except for wood density, refer to fractions and are unitless. Two traits in two furthest right columns are the properties of individual fibres. All other values are the fractions of tissues within a studied radial sector. [↑](#footnote-ref-1)
