# Supplementary material for: Fibre wall and lumen fractions drive wood density variation across 24 Australian angiosperms
Source: AoB Plants. 2013 Oct 10;5:plt046. doi: 10.1093/aobpla/plt046 (PMC4104653; doi:10.1093/aobpla/plt046)
Supplement: Additional Information [file supp_plt046_plt046supp_table3.docx]

Wood density and tissue fractions of non-vessel proportion of 24 species averaged across three replicates^[[1]](#footnote-1)^*.

| Site | Species | Wood density (g cm^-3^) | Non-vessel | Fibre | Fibre wall | Fibre lumen | Parenchyma | Axial parenchyma | Ray  parenchyma | Vessel wall | Tracheids |
| --- | --- | --- | --- | --- | --- | --- | --- | --- | --- | --- | --- |
| Cool-wet | *Allocasuarina monilifera* | 0.70 | 0.82 | 0.45 | 0.43 | 0.02 | 0.41 | 0.18 | 0.23 | 0.10 | 0.06 |
|  | *Aotus ericoides* | 0.76 | 0.90 | 0.58 | 0.57 | 0.01 | 0.37 | 0.18 | 0.20 | 0.05 | na |
|  | *Banksia marginata* | 0.66 | 0.78 | 0.52 | 0.49 | 0.03 | 0.34 | 0.15 | 0.19 | 0.13 | 0.01 |
|  | *Eucalyptus amygdalina* | 0.69 | 0.86 | 0.71 | 0.57 | 0.14 | 0.19 | 0.06 | 0.12 | 0.03 | 0.07 |
|  | *Leptospermum scoparium* | 0.87 | 0.86 | 0.69 | 0.66 | 0.03 | 0.23 | 0.07 | 0.16 | 0.06 | 0.03 |
|  | *Leucopogon ericoides* | 0.82 | 0.86 | 0.72 | 0.64 | 0.08 | 0.21 | 0.08 | 0.13 | 0.07 | na |
| Cool-dry | *Bossiaea cinerea* | 0.92 | 0.90 | 0.64 | 0.64 | 0.01 | 0.32 | 0.12 | 0.20 | 0.04 | na |
|  | *Davesia latifolia* | 0.79 | 0.83 | 0.39 | 0.35 | 0.04 | 0.38 | 0.12 | 0.26 | 0.14 | 0.09 |
|  | *Epacris impressa* | 0.80 | 0.88 | 0.52 | 0.44 | 0.08 | 0.41 | 0.18 | 0.22 | 0.07 | na |
|  | *Eucalyptus tenuiramis* | 0.84 | 0.89 | 0.70 | 0.59 | 0.11 | 0.18 | 0.06 | 0.11 | 0.03 | 0.09 |
|  | *Leucopogon ericoides* | 0.80 | 0.85 | 0.57 | 0.51 | 0.06 | 0.37 | 0.19 | 0.18 | 0.08 | na |
|  | *Persoonia juniperina* | 0.79 | 0.83 | 0.54 | 0.48 | 0.05 | 0.29 | 0.13 | 0.16 | 0.13 | 0.05 |
| Hot-wet | *Acacia mangium* | 0.47 | 0.86 | 0.62 | 0.39 | 0.23 | 0.34 | 0.27 | 0.07 | 0.04 | na |
|  | *Allocasuarina torulosa* | 0.78 | 0.79 | 0.61 | 0.57 | 0.03 | 0.27 | 0.11 | 0.16 | 0.07 | 0.05 |
|  | *Alphitonia excelsa* | 0.44 | 0.85 | 0.77 | 0.40 | 0.36 | 0.20 | 0.05 | 0.15 | 0.04 | na |
|  | *Chionanthus ramiflorus* | 0.66 | 0.85 | 0.63 | 0.46 | 0.17 | 0.31 | 0.06 | 0.25 | 0.06 | na |
|  | *Eucalyptus platyphylla* | 0.60 | 0.81 | 0.64 | 0.54 | 0.10 | 0.27 | 0.08 | 0.19 | 0.04 | 0.04 |
|  | *Ixora timorensis* | 0.62 | 0.84 | 0.56 | 0.47 | 0.09 | 0.37 | 0.05 | 0.32 | 0.06 | 0.01 |
| Hot-dry | *Acacia flavescens* | 0.85 | 0.90 | 0.77 | 0.68 | 0.09 | 0.20 | 0.12 | 0.08 | 0.03 | na |
|  | *Corymbia intermedia* | 0.78 | 0.83 | 0.67 | 0.55 | 0.12 | 0.26 | 0.11 | 0.15 | 0.05 | 0.03 |
|  | *Gastrolobium grandiflorum* | 0.86 | 0.81 | 0.58 | 0.54 | 0.03 | 0.32 | 0.09 | 0.24 | 0.10 | na |
|  | *Grevillea parallela* | 0.71 | 0.89 | 0.54 | 0.49 | 0.05 | 0.43 | 0.17 | 0.26 | 0.03 | na |
|  | *Lophostemon suaveolens* | 0.66 | 0.84 | 0.57 | 0.44 | 0.13 | 0.32 | 0.10 | 0.23 | 0.06 | 0.05 |
|  | *Persoonia falcata* | 0.74 | 0.86 | 0.71 | 0.61 | 0.09 | 0.16 | 0.08 | 0.08 | 0.06 | 0.07 |
|  |  |  |  |  |  |  |  |  |  |  |  |

.

1. * Notes: the subscript ’NV’ is omitted for brevity; all values, except for wood density, are unitless. [↑](#footnote-ref-1)
