# Supplementary material for: Fibre wall and lumen fractions drive wood density variation across 24 Australian angiosperms
Source: AoB Plants. 2013 Oct 10;5:plt046. doi: 10.1093/aobpla/plt046 (PMC4104653; doi:10.1093/aobpla/plt046)
Supplement: Additional Information [file supp_plt046_plt046supp_table4.doc]

Mean vessel area and theoretical conductivity of 24 species averaged across three replicates.

| Site | Species | Mean vessel area (µm^2^) | Theoretical conductivity  (kg m^-1^ s^-1^ MPa^-1^) |
| --- | --- | --- | --- |
| Cool-wet | *Allocasuarina monilifera* | 187 | 2.80 |
|  | *Aotus ericoides* | 487 | 2.42 |
|  | *Banksia marginata* | 329 | 4.17 |
|  | *Eucalyptus amygdalina* | 1333 | 8.96 |
|  | *Leptospermum scoparium* | 486 | 4.14 |
|  | *Leucopogon ericoides* | 286 | 2.15 |
| Cool-dry | *Bossiaea cinerea* | 499 | 2.65 |
|  | *Davesia latifolia* | 155 | 2.03 |
|  | *Epacris impressa* | 143 | 0.82 |
|  | *Eucalyptus tenuiramis* | 836 | 6.04 |
|  | *Leucopogon ericoides* | 218 | 1.57 |
|  | *Persoonia juniperina* | 229 | 2.28 |
| Hot-wet | *Acacia mangium* | 1555 | 12.13 |
|  | *Allocasuarina torulosa* | 567 | 9.81 |
|  | *Alphitonia excelsa* | 2423 | 18.84 |
|  | *Chionanthus ramiflorus* | 991 | 8.20 |
|  | *Eucalyptus platyphylla* | 1359 | 15.12 |
|  | *Ixora timorensis* | 552 | 5.15 |
| Hot-dry | *Acacia flavescens* | 1460 | 8.84 |
|  | *Corymbia intermedia* | 1078 | 13.91 |
|  | *Gastrolobium grandiflorum* | 802 | 7.82 |
|  | *Grevillea parallela* | 1528 | 10.68 |
|  | *Lophostemon suaveolens* | 446 | 5.38 |
|  | *Persoonia falcata* | 1211 | 10.18 |
